# Family poultry: Multiple roles, systems, challenges, and options for sustainable contributions to household nutrition security through a planetary health lens

**DOI:** 10.1111/mcn.12668

**Published:** 2018-10-17

**Authors:** Robyn G. Alders, Sarah E. Dumas, Elpidius Rukambile, Godfrey Magoke, Wende Maulaga, Joanita Jong, Rosa Costa

**Affiliations:** ^1^ International Rural Poultry Centre Kyeema Foundation, Maputo, Mozambique, and Brisbane Australia; ^2^ School of Life and Environmental Sciences, Faculty of Science University of Sydney NSW Australia; ^3^ Master of Public Health program, College of Veterinary Medicine Cornell University Ithaca New York USA; ^4^ Tanzania Veterinary Laboratory Agency Dar es Salaam Tanzania; ^5^ National Veterinary Directorate, Ministry of Agriculture and Fisheries Dili Timor‐Leste

**Keywords:** food security, maternal and child nutrition, nutrition‐sensitive agriculture, smallholder poultry, sustainable food production

## Abstract

Achieving sustainable production of eggs by family poultry production systems that meet both environmental health and welfare standards is a complex endeavour. Humans have been raising different species of poultry for thousands of years across many different agroecological zones. The Food and Agriculture Organization of the United Nations has identified four different family poultry production systems: small extensive, extensive, semi‐intensive, and intensive. Each of these systems varies in terms of inputs, outputs, gender dimensions, poultry health and welfare, and environmental impacts. This paper addresses key issues associated with the production of family poultry eggs in support of both improved maternal and child nutrition and sustainable, nutrition‐sensitive agricultural practices. It provides an overview of the history of poultry raising; characteristics of the different family poultry production systems; challenges and solutions to poultry production in low‐ and middle‐income countries; poultry husbandry (including breeds, nutrition, and shelter); infectious disease prevention and control in line with national and international animal health regulations; and food safety (microbial pathogens, toxins, and egg storage). To ensure that bird, human, and environmental health can flourish, it is essential for interdisciplinary research and development teams to work in collaboration with communities to ensure the long‐term environmental and economic sustainability of family poultry production enterprises that are a good fit with local circumstances.

**Key messages**

- Family poultry have been raised for thousands of years and continue to be raised in expanding numbers under a range of production systems across many different agroecological zones.
- Achieving sustainable production of eggs that meets both environmental health and welfare standards is a complex endeavour.
- Family poultry production requires attention to husbandry practices, disease prevention and control in line with national and international animal health regulations, and food safety.
- Interdisciplinary research and development is required to ensure the long‐term environmental and economic sustainability of family poultry production enterprises that are a good fit with local circumstances.

## 1 INTRODUCTION

Humans have been raising poultry for thousands of years. Archaeological evidence suggests that domesticated chickens existed in China at least 8,000 years ago with subsequent spread to Western Europe and other parts of the world by land and by sea. Domestication of chickens from the Red Jungle Fowl may have occurred separately in South and Southeast Asia. Domestic chickens appeared in Africa many centuries ago; they are now an established part of African life (Alders, 2004).

Poultry are domesticated avian species that are raised for eggs, meat, and feathers. The term poultry includes chickens, turkeys, guinea fowls, ducks, geese, and other species often considered game such as quails, pigeons, and pheasants. Chickens constitute about 90% of the poultry population and are, by far, the most important poultry species in all parts of the world (Food and Agriculture Organization, FAO, 2014). Up until the 20th century, poultry were generally raised under “extensive systems,” a term used to describe a practice where birds are largely free ranging and dependent on scavenging, with some supplementation of feed where birds were raised in larger numbers or on tracts of land where the scavenging feed resource base could not sustain them. Since the end of the Second World War, the production of poultry meat and eggs has increased dramatically due to the rapid growth of the intensive commercial poultry industry (Speedy, 2003). The selection of high yield meat and layer breeds has been shaped by agricultural value chains where commodities are priced on weight with little regard for nutrient profiles.

In relation to international development activities, a range of approaches has been employed to promote improved poultry production, including egg production, with varying degrees of success. Sustainability of small‐scale layer chicken projects has met with mixed results in peri‐urban areas and proved largely elusive beyond the end of external support in rural areas, frequently due to inadequate access to essential and affordable inputs (Alders & Pym, 2009). In unimproved extensive poultry systems, where mortality rates are high, eggs are rarely consumed by women and children, as they are preserved for hatching of replacement birds (Alders et al., 2003; de Bruyn, Bagnol, et al., 2017). Lessons learnt have been incorporated into the “Decision Tools for Family Poultry Development” manual (FAO, 2014). This FAO toolkit is designed to assist the development of feasible and appropriate family poultry projects via a stepwise decision‐making process, as a considerable proportion of development projects and programs are implemented in ecologically fragile areas where vulnerable households have to overcome poverty while also protecting the lands and natural resources on which their livelihoods depend. In addition to this manual, key reference material relating to the sustainable production of family poultry for each specific production system is listed in Table 1.

**Table 1:** Key references for essential components and history of family poultry production

	References		
Component	Extensive	Semi‐intensive	Intensive
History	Alders, 2003		FAO, 2004
Introduction to family poultry production systems	Alders & Spradbrow, 2001;	FAO, 2014	FAO, 2004, 2014
	FAO, 2014		
Roles (food, financial, and sociocultural security) of poultry including gender and livelihood strategy dimensions	Alders, 2003;	Alders, 2003;	Alders, 2003;
	FAO, 2014	FAO, 2014	FAO, 2014
Challenges and solutions to poultry development in low‐ and middle‐income countries over the past 50 years	FAO, 2010	FAO, 2010	FAO, 2010
	FAO, 2014	FAO, 2014	FAO, 2014
Poultry husbandry	Ahlers et al., 2009;	FAO, 2014	Czarick and Fairchild, 2008;
Breeds	FAO, 2010;		
Nutrition	FAO, 2014		FAO, 2004
Shelter			
Sanitation and waste management			
Infectious disease prevention and control	Ahlers et al., 2009;	Damerow, 2015;	Damerow, 2015;
National animal health regulations in relation to importation and use of veterinary pharmaceuticals (vaccines, antibiotics and vitamins and minerals)	Alders et al., 2003;	FAO, 2014	FAO, 2004
	FAO, 2014		
Food safety			
Microbial pathogens, environmental enteropathy disorder	Ahlers et al., 2009;	FAO, 2014;	FAO, 2014;
Toxins	FAO, 2014;	Zambrano et al., 2014	Zambrano et al., 2014
Egg storage under resource‐poor conditions	Zambrano et al., 2014		
Physical testing for fitness for human consumption			

In 2015, the United Nations launched the Sustainable Development Goals, a suite of goals that define key development indicators applicable to all member countries (U.N., 2015). Also in 2015, *The Lancet* and The Rockefeller Foundation launched the Planetary Health concept—the health of human civilization and the state of the natural systems on which it depends (Horton & Lo, 2015). These frameworks provide an opportunity for all development activities, including poultry development and human nutrition projects, to contribute to achieving long‐lasting positive changes both locally and globally. In relation to poultry production, as poultry are monogastric omnivores, they can potentially compete with people for the same foodstuffs (FAO, 2014). Agriculture is estimated to be responsible for approximately 10% of anthropogenic greenhouse‐gas emissions (Tubiello et al., 2015), and in relation to poultry, this includes not only the production of poultry products themselves but also all the inputs required to support this production. In terms of animal‐source food (ASF), the production of poultry eggs has been found to contribute lower levels of greenhouse‐gas emissions, with emission levels varying across production systems and opportunities for further reduction by using alternative feedstuffs (Taylor, Omed, & Edwards‐Jones, 2014).

Smallholder poultry producers commonly operate in resource‐limited situations, employing a range of activities to achieve sustainable livelihoods. Under these conditions, poultry fulfil a range of functions from income generation to strengthening social cohesion (Alders & Pym, 2009). This paper aims to highlight key issues associated with ecologically and financially sustainable smallholder poultry production and factors that must be taken into account to achieve increased egg consumption in support of both improved maternal and child nutrition and sustainable, nutrition‐sensitive agricultural practices.

## 2 METHODS

### 2.1 Review of literature

To capture as many relevant references as possible, two approaches were adopted: (a) Co‐authors involved with family poultry research and development from differing geographies and disciplines were identified, and (ii) scientific databases were searched to identify primary studies and reviews of family poultry health and production with internet search engines utilized to identify web pages that might provide references. Relevant studies, reviews, and manuals were then selected for review. Their potential relevance was examined, and nonrelevant citations were excluded. The full text of the remaining references was assessed to select publications with a primary focus on family poultry that directly related to the theme. To ensure the number of references cited was kept to a manageable number, preference was given to literature meeting the above criteria and that was also available via open access sites. References were drawn mainly from low‐ and middle‐income countries (LMICs) where family poultry play a major role in household livelihoods and nutrition security.

### 2.2 Conceptual framework

**Figure 1:**
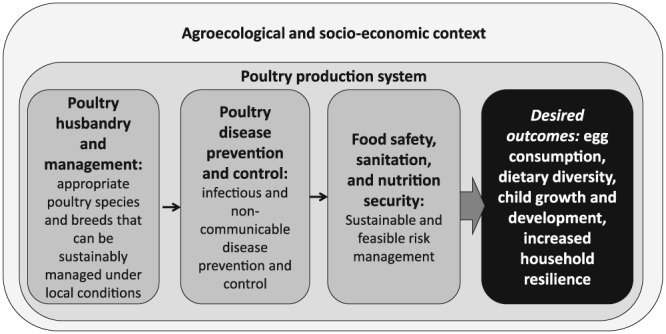
Key considerations to achieve sustainable egg production for improved maternal and child nutrition in resource‐poor settings

A conceptual framework was developed and employed to guide the structure of this paper, providing a sound foundation in relation to achieving the desired outcomes of egg consumption, dietary diversity, child growth, and development in association with family poultry production. Figure 1 illustrates the alignment of sustainable family poultry production systems with prevailing agroecological and socio‐economic conditions and appropriate management practices that include (a) selecting appropriate poultry species and breeds that can be sustainably managed under local conditions in terms of nutrition and shelter; (b) infectious and non‐communicable disease prevention and control, especially of diseases causing high mortality; and (c) risk management in terms of food safety, sanitation, and nutrition security.

### 2.3 Family poultry production systems

“Family poultry” is a term used to describe the full variety of small‐scale poultry production systems that are found in rural, peri‐urban, and urban areas of LMICs. Rather than defining the production systems per se, the term is used to describe poultry production practised by individual families as a means of obtaining food security, income, and gainful employment (FAO, 2014). The classification of poultry production systems developed by the FAO of the United Nations described in Table 2 was applied in the framing of this paper.

**Table 2:** Characteristics of the four family poultry production systems

Criteria	Small‐extensive scavenging	Extensive scavenging	Semi‐intensive	Small‐scale intensive
Production/farming system	Mixed, poultry and crops, often landless,	Mixed, livestock and crops	Usually poultry only	Poultry only
Other livestock raised	Rarely	Usually	Sometimes	No
Flock size	1–5 adult birds	5–50 adult birds	50–200 adult birds	>200 broilers
				>100 layers
Poultry breeds	Local	Local or cross‐bred	Commercial, cross‐bred or local	Commercial
Source of new chicks	Natural incubation	Natural incubation	Commercial day‐old chicks or natural incubation	Commercial day‐old chicks or pullets
Feed source	Scavenging; almost no supplementation	Scavenging; occasional supplementation	Scavenging; regular supplementation	Commercial balanced ration
Poultry housing	Seldom; usually made from local materials or kept in the house	Sometimes; usually made from local materials	Yes; conventional materials; houses of variable quality	Yes; conventional materials; good‐quality houses
Access to veterinary services and veterinary pharmaceuticals	Rarely	Sometimes	Yes	Yes
Mortality	Very High, >70%	Very High >70%	Medium to High 20% to >50%	Low to Medium <20%
Access to reliable electricity supply	No	No	Yes	Yes
Existence of conventional cold chain	No	Rarely	Yes	Yes
Access to urban markets	Rarely	No, or indirect	Yes	Yes
Products	Live birds, meat	Live birds, meat, eggs	Live birds, meat, eggs	Live birds, meat, eggs
Time devoted each day to poultry management	<30 min	<1 hr	>1 hr	>1 hr

*Note*. Source: FAO, 2014.

## 3 POULTRY PRODUCTION SYSTEMS

Family poultry can be found in all countries and play a vital role in many resource‐limited rural and peri‐urban households (Alders, 2004; Alexander, Bell, & Alders, 2004). In vulnerable households, they provide scarce ASF in the form of meat and eggs and can be sold or bartered to meet essential family needs such as medicine, clothes, and school fees. Free‐ranging village poultry are active in pest insect control, provide manure, are required for special events, and are essential for many traditional ceremonies. The output of village poultry is lower than that of intensively raised birds, but it is obtained with minimum inputs of housing, disease control, management, and supplementary feeding (Table 2).

Different ways of characterizing family poultry production have been suggested based on criteria such as size of flock, management, and purpose of production including degree of commercialization and location (FAO, 2004). For the purpose of conducting a situation analysis and planning a development intervention, FAO (2014) identified four family poultry production systems:

- small extensive scavenging;
- extensive scavenging;
- semi‐intensive;
- small‐scale intensive.

Although this range of systems may be viewed as a continuum, family poultry farmers utilize the production system that best suits their situation and objectives (FAO, 2014). Small extensive, extensive, and semi‐intensive poultry productions are common components of mixed agricultural farming systems involving crops and other livestock and permit vulnerable households to spread risks (FAO, 2014).

## 4 THE MULTIPLE ROLES OF FAMILY POULTRY

It is important to remember that family poultry fulfil multiple roles within household livelihood strategies beyond improving maternal and child nutrition. Extensively and semi‐intensively raised poultry are generally owned and managed by women and children and are often essential elements of female‐headed households (Bagnol, 2001). In many regions of the world and unlike other livestock species, women have the possibility of making the decision to sell and/or consume poultry meat and eggs without need to formally negotiate with their husband/partner (Dumas et al., 2017). This happens often in a situation in which poultry, especially chickens, are one of the only assets over which women have some degree of relative control. For this reason, chickens play an important role in women's economy and women's capacity to carry out her responsibility of caring for home and family issues (Bagnol, 2001, 2009; de Bruyn, Wong, Bagnol, Pengelly, & Alders, 2015; Dumas et al., 2017). Chickens are often considered the petty cash, that is, the smallest financial reserve, of the household, as they are sold to solve regular needs such as buying school materials, uniforms, or paying fees; going to the hospital.; buying medicine or offering a chicken to a traditional healer; and buying sugar, salt, oil, or other household items. Chickens are also extremely important for exchanging against goods, services, or to consume when there is a guest, or for rituals and ceremonies. This is particularly true when chickens are kept in small quantities at village level (Bagnol, 2009) in scavenging flocks of indigenous breeds in communities throughout low‐income, food‐deficit countries. In these settings, chickens contribute to human nutrition, livelihood, and sociocultural activities (Sonaiya, 2007). Their contributions to food availability are both direct, through supplying nutrient‐rich and culturally acceptable products for consumption, and indirect, through the sale of chickens and eggs to buy food staples, and through the provision of manure and pest insect control in association with vegetable and livestock production (Wong et al., 2017). It is common for livestock to fulfil multiple roles within households in resource‐limited settings, and livestock ownership does not necessarily translate to increased utilization of ASFs (Turk, 2013), as they may be used for sale or exchanged to fulfill other needs. However, family poultry utilization across all of these roles is high (Azzarri, Cross, Haile, & Zezza, 2014). This is due to their small size, short production cycles, and availability in most rural households, a situation that makes them more likely to be consumed, exchanged, or sold in times of need, compared with larger livestock. Chickens are particularly important in times of hunger where they are the first livestock to be sold to buy cheaper food (Bagnol, 2001) or slaughtered before higher valued animals such as pigs, goat, or cattle (Dumas et al., 2017).

It has been reported that when an activity becomes lucrative, men who previously were not involved in the activity tend to take over from women (Mayoux, 2009). Such a situation highlights that poultry development interventions may not automatically result in an improvement of women's and household's situation if subsequent increased economic benefits incentivizes men to take over flock management. In larger chicken production systems, men have tended to be in control (Sambo et al., 2015), whereas women may continue to contribute a significant portion of the required labour. Given this potential, it is critical that poultry development projects include an explicit gendered lens to avoid eroding women's control over this important livelihood activity.

## 5 SUSTAINABLE POULTRY HUSBANDRY AND MANAGEMENT

The production of poultry meat and eggs has risen dramatically over the past 50 years (Speedy, 2003), and most of this increase occurred due to the intensification of production. Although this increase has been hailed as a great success in economic terms, questions are now being asked of poultry production in terms of animal welfare (Nicol & Davies, n.d.); antimicrobial resistance (Goetting, Lee, & Tell, 2011); and the nutritional profile of poultry products (Wang, Lehane, Ghebremeskel, & Crawford, 2009).

### 5.1 Breeding and reproduction

Genetically improved specialized meat or egg‐type chickens are widely available and are used by the large majority of large‐scale commercial poultry producers and companies. These birds have been bred exclusively for meat or egg production and require high‐level nutritional and health management inputs to reach their genetic potential (FAO, 2014).

General‐purpose indigenous breed birds are raised widely in the rural regions of nearly all LMICs (Alders & Pym, 2009). In contrast with the above specialized breeds, these birds have, for the most part, considerably lower genetic potential for meat and egg production but are able to survive, reproduce, and produce meat and eggs in the often harsh, semi‐scavenging village environment. Nevertheless, there is significant variation in productivity between indigenous breeds and ecotypes across different regions, within and between countries, as well as in the climatic and nutritional environments experienced by the birds.

In addition to these two broad types, a number of dual‐purpose breeds/crossbreds are available in certain regions. These have been bred exclusively to yield relatively good meat and egg production under moderate climatic and nutritional management conditions, rather than the optimal conditions required by specialized meat and egg types. Commercial layers developed from imported parent stock have the capacity to lay more than 300 eggs per year, whereas crossbred hens lay approximately 200 eggs per year, and indigenous hens often lay only 40 to 60 eggs per year (FAO, 2004; Pym & Alders, 2016). Even when these indigenous hens are placed in laying cages and given ad libitum access to good‐quality layer diets, their laying performance is much lower than commercial layers ranging from 63 to 165 per year (FAO, 2010; Pym & Alders, 2016).

Genetic potential to produce eggs aside, a major cause of the five to eightfold difference in egg production is the time—about 13 weeks—that a broody indigenous hen spends laying and hatching a clutch of eggs and rearing the chicks to about 7 weeks of age (FAO, 2014; Pym & Alders, 2016). During the hatching and rearing time, the hen does not lay, which shortens the remaining egg production time. This means that the indigenous hen can produce about 3–4 clutches per year only. As the capacity for broodiness has been bred out of commercial‐strain layer hens (i.e., they are incapable of natural reproduction), under the right levels of lighting and nutrition, they lay continuously rather than in clutches.

To achieve a laying rate corresponding to more than 300 eggs per year, under confinement housing, a commercial layer hen requires approximately 100–110 g of a high quality layer diet, containing 11.7 MJ metabolizable energy, 180 g crude protein, and 35 g calcium per kg of weight, per day. The typical scavengeable feed resource base would provide well under half of this, which means that if reasonable productivity is required, these birds are unsuitable for unsupplemented extensive production systems. Additionally, chickens are photoperiodic and respond to daylight by timing reproduction so that it takes place at a time when feed is more likely to be plentiful, which means that, in the absence of artificial lighting, hens will lay the majority of their eggs during the spring and summer months when daylight hours are increased.

Under intensive production systems, there is a very good argument for using genetically improved meat or egg genotypes, or at least intermediate performing crossbred birds. The low productivity of indigenous breed birds, even under high‐level management and nutrition, does not warrant their use under intensive management, unless the premium paid for their eggs and meat compensates for their generally much lower performance (Pym & Alders, 2016). Due to the short duration of most development projects, the ability to influence the genetic potential of either the genetically improved egg or meat birds, or of the indigenous breed birds, is limited. Short‐term gains may be made by crossbreeding with higher producing breeds providing that all husbandry requirements to support higher productivity can be met. Longer term approaches to selecting for improved egg production traits amongst locally adapted birds are likely to yield more sustainable improvements (FAO, 2010).

### 5.2 Poultry nutrition

Proper nutrition is essential for flock health, survival, and productivity. Poultry are monogastric omnivore animals requiring at least 38 nutrients in proper balance, and this balance varies by poultry type, genetic strain, body size, and age, as well as the ambient temperature, level of physical activity, and presence of stressors (e.g., disease; Klasing, 2016). In addition, egg quality is influenced by certain nutrients and dietary feed formulation with insufficient or excessive nutrients in feed leading to poor‐quality eggs (Wang, Yue, Wu, Zhang, & Qi, 2017). It is important to note here that the nutritional content of eggs listed in national food composition tables in LMICs is often imported from U.S. or U.K. databases and so may not accurately reflect the local situation (de Bruyn et al., 2016).

The National Research Council (United States) has published the minimal nutrient requirements for egg laying hens derived from the literature (Table S4; National Research Council, 1994; Leeson, 2011). Compared with other poultry, the calcium requirement for laying hens is particularly high to meet the demands of eggshell development. This is especially true for older or high‐producing hens, who require additional dietary calcium to maintain eggshell strength (Klasing, 2016).

For extensively raised indigenous chickens, these nutritional requirements are primarily met by scavenging, coupled with occasional supplementary feeding of home‐grown grains and household food waste. Feed is an important component in sustainable egg production enabling the supplemented chickens to produce more eggs than chickens surviving solely on the scavenging feed resource base (Goromela, Kwakkel, & Verstegen, 2008). Although nutritionists design complete rations to meet a laying hen's nutritional requirements determined by maintenance, body weight, and level of egg production (Leeson, 2011), in scavenging systems, the energy, protein, and micronutrient content of the feed is often critically deficient, especially during the dry season in tropical zones when feed resources are scarce. A study by Goromela et al. (2008) in Tanzania revealed that scavenged feed resources consumed daily by free‐ranging chickens vary from 45 g in the dry season to 54 g in the rainy season, amounts considered insufficient to fulfill the protein requirements for high egg production.

In contrast, because poultry raised in intensive systems are necessarily housed, they need to be provided with balanced feed. Commercial feeds are formulated to meet the nutritional requirements of a particular type of bird at a particular stage of maturity (e.g., starter, grower, and layer feed) and are available with different contents of protein and micronutrients. Commercial feeds are therefore ideal for meeting the nutritional requirements of the flock, especially for a new poultry producer.

However, for small‐scale producers in low‐income countries, especially those in rural areas, feed access and cost can be a major constraint to productivity and economic sustainability of the enterprise (FAO, 2014). In a semi‐intensive egg‐production program in rural Zambia, for example, feed access was limited by erratic stocking of commercial layer mash by local shop owners, impassable roads during the rainy season, and lack of transportation (Dumas, Lungu, Mulambya, Lewis, & Travis, 2018). As a result, producers were at times forced to feed only maize bran, leading to dramatic drops in egg production (Dumas et al., 2018). Conditions for successful interventions involving feed provision are outlined in Table 3.

**Table 3:** Examples of successful extensive and semi‐intensive poultry management interventions in rural areas, including country, time period, nature of interventions, outcomes, and conditions for success

Country	Production system	Program start	Program end	Nature of interventions	Outcomes	Conditions for success	References
Mozambique and Tanzania	Extensive	July 2002	November 2005 (approach is ongoing with vaccination coverage increasing in both countries)	Project scale: Mozambique—45 villages across five provinces; Tanzania—10 villages across two regions	70–500% increased flock size across project sites, improved off‐take* and reduced mortality	Gender‐sensitive approach	Alders, 2009; Harun et al., 2009; Msami & Young, 2009
				Community vaccination against ND through trained community vaccinators	Consumption and sale of chickens increased significantly	Cost‐sharing mechanism with farmers paying a fee‐for‐service to community vaccinators and community vaccinators purchasing vaccine	
				Participatory implementation of vaccination program	Small increase in consumption and sale of eggs	Capacity building of and coordination with community members, NGO and government workers, community leaders, etc.	
				Training in general poultry husbandry for extensive systems		Development of appropriate extension materials	
				In‐country thermotolerant ND vaccine production		Timely availability of ND vaccine	
Malawi, Mozambique, Zambia, and Tanzania	Extensive	2009	2013	Project scale:	More than 48.8% of households vaccinating regularly	As above.	Fisher, 2014
				Mozambique – 33 villages; Tanzania—27 wards Zambia: eight villages	Chicken off‐take 13.7% in Mozambique, 15.4% Malawi, and 33.9% in Tanzania		
				Malawi: 50 villages	Increase in flock size 10.1% in Mozambique, 9.9% Malawi, and 20.9% in Tanzania		
				Thermotolerant ND vaccine production			
				Community vaccination against ND through trained community vaccinators			
				Participatory implementation of vaccination program			
				Training in general poultry husbandry for extensive systems			
Tanzania	Extensive	April 2014	Ongoing	Project scale: 12 villages across two districts	Increased flock size	Participatory and cost‐sharing approaches to implementation of ND vaccination program	de Bruyn, Bagnol, et al., 2017; de Bruyn, Thomson, et al., 2017
				Community vaccination against ND through trained community vaccinators	Increased participation in vaccination campaigns, especially among households having larger flocks	Timely availability of ND vaccine	
				Participatory implementation of vaccination program		Children significantly more likely to consume eggs if mother also consumed	
				Training in general poultry husbandry for extensive systems			
Zambia	Extensive	July 2007	Ongoing (evaluation endline: November 2011)	Project scale: >5,271 farmers in one province	160% increase in average flock size in participating HHs compared with no change in controls	Requires a supporting organization with local relationships and capacity, including refrigeration for vaccine	Dumas et al., 2016
				Community vaccination against ND	65% increase in poultry profitability in participating HHs		
				Training of “Poultry Lead Farmers” and formation of community poultry production groups	No change in chicken meat or egg consumption in participating HHs		
				Training and demos for an improved model of poultry housing			
				Training in supplementary feeding and disease prevention			
Zambia	Semi‐intensive egg production	June 2010	June 2016	Project scale: 24 villages across two districts	Pilot: >11,000 eggs were produced and locallysold in 10 months	Requires a supporting organization to provide access to replacement layers, a viable local market for eggs, and local access to layer feed	Dumas et al., 2016; Dumas, 2017; Dumas et al., 2018
				Trained and supported households or small groups in semi‐intensive egg production practices and business management	Nearby HHs reported a 75% increase in egg consumption		
				Provided 40 layers and materials for secure poultry housing	Egg producers had a 45% increase in total HH income		
					Extended pilot: >156,000 eggs were produced and locally sold in 12 months		
					Young children 6–36 months were significantly more likely to eat eggs if they lived near an egg production centre		
					March/April 2018: 8915 poultry owners (65% female), 34,196 chickens vaccinated across 4 districts

*Note*. ND: Newcastle disease; HH: Household.

* Off‐take defined here as eaten by household, sold, exchanged, given to guests or eaten in association with ceremonies.

As an alternative, home‐mixed feeds can be formulated using locally available grains, protein‐rich feedstuffs, and a vitamin/mineral premix (Damerow, 2015; FAO, 2004; Table S5). Primary energy sources are grains, grain by‐products, and vegetable and animal fats (Chiba, 2009). Amino acids, often the most challenging and costly nutrients to provide in smallholder systems, are primarily derived from soybean meal and fish/meat meal or their alternatives (Chiba, 2009). Along with the vitamin/mineral premix, added ground limestone oyster shells provide additional calcium, whereas bone meal or rock phosphate provide added phosphorus (Chiba, 2009), both critical for eggshell development.

Because many of the components of these feeds are also suitable for human consumption, efforts should be made to utilize alternatives to avoid competition between humans and poultry for feedstuffs, particularly in food‐insecure communities (FAO, 2014). By‐products from local crop processing (brans, oils, and meals) can partially fulfill the energy and protein requirements of poultry (FAO, 2014). For example, a by‐product of starch production, 15% cassava pulp, can replace maize in layer diets with no detrimental effect on egg production or quality, with the exception of paler egg yolks (Iji, Bhuiyan, Chauynarong, Barekatain, & Widodo, 2011). Termites, maggots, or earthworms can be cultivated or collected using traps and used as suitable protein sources, whereas blood can be dried on a vegetable carrier to make blood meal (FAO, 2014). Eggshells, which are 98.2% calcium carbonate, can be boiled, dried, and crushed and provided as a microbially safe substitute for limestone (Gongruttananun, 2011).

Many of these alternatives are also suitable to provide to extensively raised, indigenous chickens to supplement their scavenging resources, thereby optimizing body weight and fat deposition necessary for maximal egg production (Ahlers et al., 2009; FAO, 2014).

Additionally, there are numerous lesser known crops and wild plants that are well adapted to particular agroecological conditions that may be appropriate livestock feed resources (Quansah & Makkar, 2012), but further research is needed to examine their suitability as poultry feed. The exact nutritional content of these alternatives are rarely known, and dietary fibre or antinutritive factors may inhibit nutrient bioavailability and negatively affect egg production (Martens, Tiemann, Bindelle, Peters, & Lascano, 2012).

### 5.3 Shelter

Housing and other infrastructure requirements vary considerably depending on the production system concerned. For all poultry systems, the basic requirements for poultry housing are space, ventilation, light, and protection.

In an extensive system—which typically rely on scavenging as the primary feed resource—birds must remain free ranging during the day but can be housed at night. Predators are often a major challenge, especially in rural areas, and chicks are particularly vulnerable (Alders & Pym, 2009). Sturdy, elevated poultry houses built using locally available materials can reduce the risk of predation and additionally serve to concentrate faeces (to be used as fertilizer), protect the flock from adverse weather and theft, and facilitate health inspections and vaccinations (Ahlers et al., 2009). Care must be taken to use designs and materials that do not promote infestations of internal and external parasites; the design should allow for good ventilation and easy cleaning to prevent the transmission of infectious disease agents within the flock.

By definition, intensively and semi‐intensively raised flocks require permanent housing. These must be designed with a ventilation system to maintain optimal temperature in hot climates (Czarick III & Fairchild, 2008); use good quality building materials (FAO, 2004); and consider biosecurity practices.

## 6 DISEASE PREVENTION AND CONTROL

### 6.1 Infectious diseases

Infectious diseases are recognized as one of the major constraints to improving family poultry production (Pym & Alders, 2016). Viral diseases have a major impact on the health and productivity of poultry. The major tools that poultry owners have to protect their flocks against these diseases are good biosecurity and vaccination. Although there is no specific treatment for viral diseases, broad‐spectrum antibiotics may only be of some benefit to prevent or treat secondary bacterial infection. Numerous studies have identified Newcastle disease (ND) as the major killer disease of chickens globally.

ND is a highly contagious viral infection that affects many species of domestic and wild birds. Chickens, turkeys, pigeons, and parrots are most susceptible, whereas a mild form of the disease affects ducks, geese, pheasants, quail, and guinea fowl. ND is a member of the Paramyxoviridae family of viruses, which also includes the human measles virus. The pathogenesis and epidemiology of ND was reviewed by Alexander et al. (2004). The major source of infection of ND is the introduction of new birds to family poultry flocks. Markets also serve as a common source of ND infection, sometimes through the random sale of infected birds during outbreaks to salvage those not yet showing clinical signs (Ahlers et al., 2009). Models for the sustainable control of ND under resource‐limiting conditions through the training of community vaccinators who work on a fee‐for‐service basis have proved sustainable in Sub‐Saharan Africa since the early 2000s (Alders et al., 2003; Alders, Bagnol, & Young, 2010; Alexander et al., 2004; Dumas et al., 2016; Table 3). Vaccination of family poultry has received little attention from animal health services in Sub‐Saharan Africa with most funding from national governments and donors continuing to focus on ruminants. The introduction of cost‐sharing methodologies using community vaccinators has facilitated increased coverage of vaccinations against ND in family poultry in rural areas (Alders, 2009; Alders et al., 2003).

However, family poultry are exposed to a number of other viral pathogens such as avian influenza, fowlpox, infectious bronchitis, infectious bursal disease, and Marek's disease, all of which can cause significant mortality and morbidity (Ahlers et al., 2009).

Bacterial diseases may also have a significant impact on the health and productivity of family poultry. Poultry owners have a number of tools to protect their flocks against these diseases: good biosecurity, treatment with specific antibiotics, and vaccination. Chronic respiratory disease, colibacillosis, fowl cholera, fowl typhoid, infectious coryza, and pullorum disease have been recorded in extensively raised indigenous chickens. Salmonellosis (Ahlers et al., 2009), pasteurellosis, and mycoplasma infection occur across all production systems.

Concerns over growing antimicrobial resistance in association with the inappropriate use of antibiotics in food animals have led to increasing regulation of their use. Additionally, drug residue in eggs is of concern due to the protracted nature of egg development, and a review of the literature has found very large variation in the duration of persistent detectable residue of most antimicrobials in eggs (Goetting et al., 2011). As a result, few antibiotics are approved for laying hens in either the United States or the European Union (Marmulak et al., 2015), and any extra‐label antimicrobial use should occur under the supervision of a veterinarian. Without the guidance of a veterinarian or livestock officer, few family poultry owners are likely to be aware of egg withholding time (the time after administration of the antimicrobial during which eggs should be discarded) appropriate for the drug, dose, route, and duration of treatment.

Parasitic diseases, mycotoxins, and nutritional deficiencies may also have an impact on productivity of family poultry. The impact of parasitic diseases including helminths, ectoparasites, and coccidia (Ahlers et al., 2009) has also been demonstrated.

Disease in hens, as well as poor husbandry or nutrition, may affect egg production. A hen in poor condition will produce fewer or even no eggs. The quality of eggs can also be affected by several diseases and disorders (Ahlers et al., 2009).

Where poultry disease surveillance and diagnosis are weak, participatory epidemiology (Alders & Spradbrow, 2001) can be employed to identify diseases or disease syndromes of importance, which can be confirmed by laboratory diagnosis.

### 6.2 Biosecurity

The FAO and the World Organization for Animal Health (OIE) define biosecurity as the implementation of measures to reduce the risk of the introduction and spread of disease agents. Although ways of classifying these measures vary, the two basic principles are bioexclusion (i.e., preventing infectious agents from entering the farm by introducing healthy birds and providing clean supplies of feed, water, and litter) and biocontainment (i.e., preventing infectious agents from spreading) and involve segregation of the flock (confinement, controlling contacts with other birds and/or people, and introduction of healthy birds only); cleaning (shelters, equipment, clothes, and shoes); and disinfection (FAO, 2014). Specific recommendations for family poultry settings found in literature usually refer to highly pathogenic avian influenza‐related risks and vary according to production system (FAO, 2014). It is important to note that investing in adequate biosecurity practices is commonly difficult for small‐scale intensive poultry producers with low profit margins, which places them at risk, as the frequent movement of inputs and outputs increases the opportunity for disease introduction and spread.

### 6.3 Diet‐related diseases

In areas where the scavenging feed resource base is limiting, non‐communicable diseases related to poor nutrition, for example, protein and/or vitamin deficiencies, may occur seasonally (FAO, 2004). In situations where commercial poultry rations are not routinely tested, deficiencies of key nutrients may also occur in these rations with their absence being detected only when birds fail to grow or produce as expected or become immunologically compromised and susceptible to an increased range of infectious diseases.

### 6.4 Ethno‐veterinary medicine

Rural and family poultry systems in LMICs typically lack access to organized poultry health inputs, and where they do exist, farmers are usually constrained by lack of finance and unavailability of consultancy advice from veterinary and extension officers. Small flock size, mixed‐age and species flock composition, improper housing, scavenging, among other factors have made the use of conventional schedule‐oriented health inputs like medication and vaccination difficult. Conventional poultry health packages are designed for the commercial sector and therefore feature large dose‐packages usually for hundreds or thousands of birds. Hence, in the villages, farmers usually rely on traditional medicine for meeting health care needs (Alders & Spradbrow, 2001). The application of indigenous knowledge to treat animal diseases is known as ethno‐veterinary medicine and is defined as an indigenous animal health care system that includes the traditional beliefs, knowledge, skills, methods, and practices of a given society. The active ingredients of some traditionally used treating plants may contain many compounds effective against different clinical signs or may just alleviate signs found across illnesses such as pain and are therefore not specific treatments to any one particular disease.

Making time to document ethno‐veterinary practices provides an opportunity to increase understanding of community perceptions regarding the origins of disease and how it may be controlled.

### 6.5 Toxins

Aflatoxin, the most potent and widespread mycotoxin, has been associated with increased incidences of liver cancer in adult humans and reduced growth rates and stunting in infants and children in LMICs from consumption of contaminated dietary staples, particularly maize and groundnuts (Strosnider et al., 2006). Equally, poultry exposure to aflatoxin‐contaminated feed will lead to poor feathering, listlessness, anorexia with lowered growth rate, poor feed utilization, decreased weight gain, decreased egg weight and production, increased susceptibility to environmental and microbial stresses, and increased mortality (Ortatatli, Oguz, Hatipoglu, & Karaman, 2005), causing severe economic losses in the poultry industry. Hence, strategies that aim at reducing grain mycotoxin contamination such as proper harvesting, drying, and storage may help mitigate significant health problems and production losses in poultry and potential exposure of humans to the toxin (more likely via the consumption of contaminated poultry liver than eggs; Sineque, Macuamule, & dos Anjos, 2017).

### 6.6 National and international animal health regulations

Animal health practices are governed by national and international regulations. The purchase and use of veterinary pharmaceutical agents, such as antibiotics and vaccines, are proscribed by law to ensure the appropriate use of these products. For example, veterinary pharmaceutical should be appropriately registered in the country where they are to be administered, and the administration of antibiotics and vaccines, especially where needles are used, should be done by authorized technicians.

## 7 FOOD SAFETY, SANITATION, AND NUTRITION SECURITY

### 7.1 Zoonotic pathogens and risks to human health

Poultry production has received increasing attention from the public health community in recent years due to its links to direct transmission of zoonotic diseases to humans through contact with poultry, or indirect transmission through poultry food products or waste. Zoonotic diseases of major interest include salmonellosis, campylobacteriosis, colibacilosis, and highly pathogenic avian influenza. These diseases have been more problematic in intensive production systems; however, their prevention (e.g., by purchasing birds from flocks certified free of key diseases, training on appropriate biosecurity, and hygiene practices) should be included in any new family poultry project, irrespective of the production system (FAO, 2014). For example, *Salmonella* is of particular public health concern, and contamination of eggs can be limited through good management, such as routine disinfection of poultry housing between flocks and pest eradication (Whiley & Ross, 2015).

In addition to the risk of clinical disease, family poultry ownership has been negatively linked with child nutrition outcomes because of its potential to contribute to an unsanitary household environment (Gelli et al., 2017), leading to increased exposure of household members to chicken faeces and feather dust. In extensive systems, although the waste produced is minimal, there is high human–chicken interaction, and infants may consume chicken faeces or contaminated dirt during exploratory play (Ngure et al., 2013). Exposure to livestock (Zambrano, Levy, Menezes, & Freeman, 2014); geophagy (George et al., 2015); animal faeces in the compound (Headey et al., 2017); and corralling livestock inside (Headey & Hirvonen, 2016) has been statistically associated with environmental enteric dysfunction (EED)—a disorder associated with reduced intestinal absorptive capacity and undernutrition—diarrhoea, and stunting in some but not all countries studied. In rural Ethiopia, poultry ownership was positively associated with linear growth, but corralling poultry indoors was negatively associated with linear growth, completely off‐setting the benefit of poultry ownership on child nutrition in those households (Headey & Hirvonen, 2016). However, in a longitudinal study conducted in central Tanzania over 2 years, no significant association was observed between keeping indigenous chickens within human dwellings overnight and linear growth performance or diarrhoeal incidences in 503 children under 5 years (de Bruyn, 2017). Research continues in this same study site with an analysis of findings over 4 years to become available by the end of 2018. The complexity of potential linkages between EED pathways and child stunting was emphasized in a recent systematic review (Harper, Mutasa, Prendergast, Humphrey, & Manges, 2018). Key findings by Harper et al. (2018) were that it is possible that EED is not a single entity, but instead a set of phenotypes dependent on unique environmental exposures that vary geographically; that it is not firmly established that EED is always consequential to linear growth, or indeed that it is definitively associated with stunting; and that some support existed for the link between intestinal inflammation and stunting.

Given these potential risks, appropriate housing and waste management practices are vital to ensure that poultry can contribute to improved nutrition outcomes. Birds must be provided appropriate shelter as described above, to minimize their negative impact on household hygiene and the local environment. For chickens raised in extensive systems, a lack of durable poultry housing to resist predators, adverse weather, and theft incentivizes owners to shelter birds inside the family home at night (Msami, 2008). Emphasis should be placed on educating farmers about the potential health risks of this practice and how to instead build sturdy, elevated poultry houses for nighttime sheltering of birds (Ahlers et al., 2009).

In semi‐intensive or intensive poultry systems, where larger numbers of birds are sheltered in permanent housing, strict biosecurity practices (e.g., designated footwear, exclusion of children from the poultry house, and hand‐washing with soap before and after entering the poultry house) can prevent contamination of the household environment. These systems additionally require a waste management plan, particularly during the rainy season when microbial count in surface water increases due to run‐off from areas contaminated by livestock faeces (Chouhan, 2015). Application of poultry litter to agricultural lands as an organic fertilizer is a safe, sustainable disposal method suitable for mixed crop‐livestock systems, particularly if it is first composted to inactivate potentially harmful pathogens and produce more stable organic matter (Kelleher et al., 2002).

### 7.2 Egg storage and quality assessment in resource‐limiting conditions

Between 8% and 24% of raw eggs in Africa have been reported to be contaminated with *Salmonella*, and proper storage, handling, and preparation are therefore critical to their safe consumption (Ejo, Garedew, Alebachew, & Worku, 2016). With fertile eggs, the embryo will start to grow even at ambient temperature (above 20°C). Eggs should therefore be kept in a cool, shady place. Where refrigeration is not available, a basket or box containing sawdust or bran placed in a hole in the floor in the coolest part of the dwelling makes a good system for storing eggs. Eggs for incubation should not be stored for longer than 2 weeks (Ahlers et al., 2009). Egg storage conditions are important, especially if a more consistent supply of eggs is to be achieved via seasonal production of eggs by indigenous or dual‐purpose hens.

The quality of albumen declines very rapidly when eggs are stored at room temperature, especially in hot climates. Refrigeration is effective in maintaining quality for several months. Oiling eggs on the day of lay will preserve their quality for several weeks, and the oil film also prevents germs from entering (Ahlers et al., 2009). Fresh eggs can be distinguished from old ones by the height of the albumen (the white or clear part of the egg) once an unboiled egg is opened and put on a dish (Ahlers et al., 2009).

Hard‐boiled eggs can be stored for several weeks. These eggs might also be oiled to preserve their quality for even longer periods. Another possibility is to store raw eggs in waterglass (sodium silicate) solution. Eggs will keep for several months in waterglass if covered and stored in a cool place. The waterglass solution is made by mixing one part of waterglass (sodium silicate) to five parts of previously boiled but cooled water (Ahlers et al., 2009).

## 8 FAMILY POULTRY AND MATERNAL AND CHILD NUTRITION: CHALLENGES AND SOLUTIONS

Sustainable, food‐based approaches to improved maternal and child nutrition will vary according to local conditions. In urban and peri‐urban areas, it may be that promoting the purchase and consumption of commercially produced chicken eggs represents the optimal benefit‐cost investment. In rural areas where the inputs required to support efficient and humane intensive production of chicken eggs are not readily available, consideration can be given to supporting semi‐intensive and extensive chicken production. These systems may contribute to nutrition security directly and indirectly through the sale and home consumption of chickens and eggs at both the household and community levels.

In many rural areas, farming households are reluctant to eat surplus chickens or eggs, and in some regions, the consumption of eggs is prohibited for children and women by tradition (Alders et al., 2003). As mentioned above, the conservation of eggs and the hatching of chickens are important in situations of high chicken mortality, where replacement birds are essential. Following the introduction of effective improved family poultry production programs, it can take up to 2 years for households to feel confident that their poultry will no longer die in large numbers enabling them to consume poultry and poultry products in increased quantities (Harun et al., 2009). Additionally, in some communities despite efforts to improve maternal diet quality during pregnancy, the ability to influence maternal diets goes well beyond food availability. In many locations, maternal and child undernutrition is accompanied by inadequate obstetrical support services, a situation which contributes to customs recommending the avoidance of foods, such as eggs, which could lead to increased birth weight and obstructed labour (Arzoaquoi et al., 2015). If the benefits of the consumption of eggs and other ASFs are to be fully realized during the 1,000‐day window of opportunity, then interventions that enable women to make sound dietary choices during pregnancy are essential.

## 9 CONCLUSIONS

For thousands of years, poultry raising has been, and continues to be, a significant component of human civilization with differing breeds and production systems arising in association with local cultures and agroecological systems. To ensure that bird, human, and environmental health can flourish, it is essential for interdisciplinary research and development teams to work in collaboration with communities to ensure the long‐term environmental and economic sustainability of family poultry production enterprises that are a good fit with local circumstances.

Achieving sustainable improvements to household nutrition security in resource‐poor settings is a major challenge, as rural households frequently face multiple issues from extreme poverty to environmental degradation. Consequently, attaining household nutrition security requires a multipronged approach that is feasible in the long‐term under local conditions and which may include improved family poultry production that provides increased numbers of birds and eggs for sale as well as home consumption; improved linkages between family poultry producers and public and private animal health service providers; and nutrition education targeting men and women.

## CONFLICTS OF INTEREST

The authors declare that they have no conflicts of interest.

## CONTRIBUTIONS

RA led the conceptualization and preparation of the review paper with contributions from SED (conceptualization, primary authorship of sections on poultry nutrition, poultry shelter, and risks to human health); ER (sanitation and food safety); GM (food safety and toxins); WM (poultry nutrition); JJ (animal health regulations); and RC (infectious disease and biosecurity). All authors contributed to and approved the final manuscript.

## Supporting information


**TABLE S1** Requirements of laying hens for select nutrients at 100 g feed/hen/day*
**TABLE S2.** Balanced home‐mixed ration for layer hens
