# Supplementary material for: Family poultry: Multiple roles, systems, challenges, and options for sustainable contributions to household nutrition security through a planetary health lens
Source: Matern Child Nutr. 2018 Oct 17;14(Suppl 3):e12668. doi: 10.1111/mcn.12668 (PMC6221142; doi:10.1111/mcn.12668)
Supplement: Supplementary file 1 — TABLE S1 Requirements of laying hens for select nutrients at 100 g feed/hen/day* TABLE S2. Balanced home‐mixed ration for layer hens [file MCN-14-e12668-s001.docx]

**Supplementary material**

**TABLE S1** Requirements of laying hens for select nutrients at 100 g feed/hen/day*

|  | White-egg layers | Brown-egg layers |
| --- | --- | --- |
| Crude protein, % | 15.0 | 18.0 |
| Arginine, % | 0.70 | 0.85 |
| Lysine, % | 0.69 | 0.84 |
| Methionine, % | 0.30 | 0.36 |
| Methionine + cystine, % | 0.58 | 0.71 |
| Threonine, % | 0.47 | 0.57 |
| Tryptophan, % | 0.16 | 0.19 |
| Calcium, % | 3.30 | 4.00 |
| Phosphorus, available, % | 0.25 | 0.30 |

** Based on dietary metabolized energy of approximately 2,900 kcal/kg and an assumed rate of egg production of 90% (90 eggs per 100 hens daily)*

*Source:* (National Research Council, 1994)

**TABLE S2** Balanced home-mixed ration for layer hens

|  | Amount, in kg, for 45 kg of layer ration (% total) |
| --- | --- |
| Coarsely ground grain (maize, wheat, rice, millet, sorghum, etc.)* | 24.0 (53.3%) |
| Wheat bran, rice bran, etc. | 7.5 (16.7%) |
| Soybean meal, peanut meal, cottonseed meal (low gossypol), safflower meal, sunflower meal, sesame meal, etc.** | 6.7 (14.9%) |
| Meat meal, fish meal, soybean meal** | 1.3 (2.9%) |
| Alfalfa meal | 1.8 (4.0%) |
| Bone meal, rock phosphate | 1.0 (2.2%) |
| Commercial vitamin/mineral premix*** | + |
| Ground limestone, oyster shell, or eggshell | 1.3 (2.9%) |

* Cassava or cassava pulp, sweet potato, coco yam, and arrow root may also be used as suitable feed energy sources (FAO, 2014)

** Alternatives include earthworm meal, maggot meal, winged bean, pigeon pea, jack bean, and leaf protein concentrates such as *Moringa oleifera* (FAO, 2014)

*** Salt should be added (200 g) if not included in the vitamin/mineral premix
